# The Superficial Venous System of the Forelimb of the Anubis Baboon (*Papio anubis*): The Distribution of Perforating Veins and Venous Valves

**DOI:** 10.1155/2019/3147439

**Published:** 2019-10-07

**Authors:** Robert Haładaj, Karolina Barszcz, Michał Polguj, Mirosław Topol

**Affiliations:** ^1^Department of Normal and Clinical Anatomy, Medical University of Lodz, Poland; ^2^Department of Morphological Sciences, Faculty of Veterinary Medicine, Warsaw University of Life Sciences — SGGW, Poland; ^3^Department of Angiology, Medical University of Lodz, Poland

## Abstract

The superficial veins of the forelimb show high variability, both in man and in other primates, regarding the number of main venous trunks, their course, as well as the origin and location of openings. The distinction between two venous systems–the superficial and deep was made based on the relation of specific venous channels to the deep fascia; both groups of veins anastomose to each other through perforators piercing the deep fascia. In our work, we paid special attention to the organization of the venous system within the forelimb of the Anubis baboon (*Papio anubis*), as well as communications between the superficial and deep venous system. The main aim of the study was a detailed examination of the location of venous valves and perforating veins in forelimb of Anubis baboon. In the Anubis baboon, we observed the absence of the basilic vein. The main vessel within the forelimb, in the superficial venous system, was a well-developed cephalic vein. In all the cases, the cephalic vein opened into the external jugular vein. Also, in all of the examined specimens, there was an additional anastomosis connecting the cephalic and external jugular vein, i.e., persistent jugulocephalic vein located anterior to the clavicle. The venous vessels in the Anubis baboon were arranged in two main layers: superficial and deep, with both systems being connected by perforators located at the level of the carpus and cubital fossa. The number of venous valves within the cephalic vein was greater on the forearm the same as the mean intervalvular distance.

## 1. Introduction

The system of superficial veins of the primates' forelimb (thoracic or upper limb) shows two basic types of arrangements dependent on the number of main venous channels present in the superficial venous network. Typically, in humans and orangutans, two major venous blood vessels, i.e., the cephalic and basilic vein respectively, are observed on the lateral and medial side of the upper limb [1]. However, in the case of other primates, there is a single main venous trunk within the upper limb, homologous to the cephalic vein, defined by some authors as the lateral vein. Both systems anastomose with each other at many points through the perforating veins piercing the deep fascia [1–7].

Detailed knowledge on the role of perforating veins and various limb venous valves has increased significantly over the past few decades. In relation to humans, research in this area is focused to a large extent on clinical issues: etiology and surgical treatment of lower extremity varicose veins, role of veins in the design of flaps based on their vascularization, upper limb replantations in reconstructive surgery, and venous grafting [6, 8–14]. In the field of comparative anatomy of the forelimb veins of primates, in addition to the general anatomical descriptions, we found only a few studies on the distribution of perforating veins and venous valves in the upper limbs [1]. While analyzing data from the literature, we drew attention to the fact that in the field of research on the venous system of the limbs, the data mostly concerns the veins of lower limbs of humans.

However, many issues concerning the distribution of perforating and venous valves in the upper limbs in various non-human primates and mammals still require explanation; the description of which venous channels are connected by the individual perforators needs supplementation due to the lack of detailed data available. Very few papers were published on this topic [1]. Moreover, we did not find a study on nonhuman primates, by which the differences in the distribution of venous valves between the arm and the forearm were analyzed statistically.

The Anubis baboon was chosen because of the unique possibility of carrying out anatomical research on such a rare research material. It is a model representing the upper limb venous pattern characterized by the presence of a single main stem—the cephalic vein [15, 16]. In our work, we paid special attention to the organization of the venous system within the forelimb (thoracic limb) in the Anubis baboon, as well as communications between the superficial and deep venous system. The main aim of the study was a detailed examination of the location of venous valves and perforating veins in forelimb of Anubis baboon. The differences in venous valves distribution between the arm and forearm were analyzed statistically to check the relation between the location (arm or forearm) and the number of venous valves and distances between those valves. Particular attention was paid to the detailed description of the link between the individual perforating veins and the deep veins. An attempt was also made to analyze possible directions of the blood flow between the superficial and deep venous system. The differences in the superficial system of baboon, human, and other primates were also discussed.

## 2. Material and Methods

Our studies were carried out on 10 (8 male and 2 female) corpses of adult Anubis baboons (*Papio anubis*), fixed in 10% formalin solution. The study was approved by the Local Ethical Committee on Animal Testing (approval no. 33/ŁB 552_DNT/2011). No live animals were used for the purpose of this research and no animals were killed for the purpose of this study. The corpses of dead animals were obtained from the Zoo of Lodz (for over fifteen years) and stored as a part of an anthropological collection in our Department of Normal and Clinical Anatomy. Finally, twenty thoracic limbs were dissected. The dissection was carried out in a stratigraphic manner, according to previously described protocols [17–22]. After removal of the skin and subcutaneous tissue, the course and arrangement of the superficial veins were examined. At this stage of the procedure, the assessment of the distribution of perforating veins between the superficial and deep veins was performed. Subsequently, measurements of the external diameters of main venous channels and perforating veins were made with a Digimatic digital caliper (Mitutoyo, Kanagawa, Japan). Each measurement was performed twice—the average of both measurements was the final result.

After that, the deep dissection of the carpal region and the cubital fossa was conducted to trace the course of the main perforators. At this stage, the superficial flexor compartment was separated, cut, and reflected. After detailed examination of location of the perforating veins, the main venous channels were cut alongside (in the direction of the long axis of the specific vessel) by using microsurgical scissors to trace the distribution of the venous valves. The distribution of the valves was assessed in situ, under magnification obtained by using a Heine HR 2.5X high-resolution binocular loupe (Heine Optotechnik, Herrsching, Germany). The intervalvular distances were measured in situ, both on the arm and on the forearm [5, 21]. Distances between the orifice of main cubital perforator and adjacent valves were also measured. The number of valves in the cephalic veins respectively in the forearm segment and in the arm one of the cephalic veins was compared by statistical analysis. Correlations between the corresponding results on the arm and forearm, as well as on the left and right sides were evaluated. Distribution normality of the variables was assessed with the Shapiro–Wilk test. Despite the small size of the sample, deviation from distribution normality was noted only for the intervalvular distances. This has made it possible to use parametric methods for assessment of significance of differences between the groups (Student's *t*-test) for the majority of variables. For variables with deviations from the normal distribution, the nonparametric Mann–Whitney test was applied. The next stage has consisted in the assessment of correlations between the variables with a specific correlation coefficient: Spearman's rho. The significance level adopted in the analysis is *p* < 0.05. The calculation was made with IBM SPSS Statistics 21.0. In our work, we applied current anatomical terminology, in justified cases, referring to the older terminology used in the cited works.

## 3. Results

### 3.1. Hand and Forearm

Superficial veins of the forelimb in Anubis baboon (*Papio anubis*), similarly formed to humans and other primates; two systems—superficial and deep. Both systems were separated by a deep fascia and connected through numerous perforating veins. The main venous channel in the superficial venous system of the Anubis baboon was the cephalic vein, while the basilic vein was absent.

**Figure 1:**
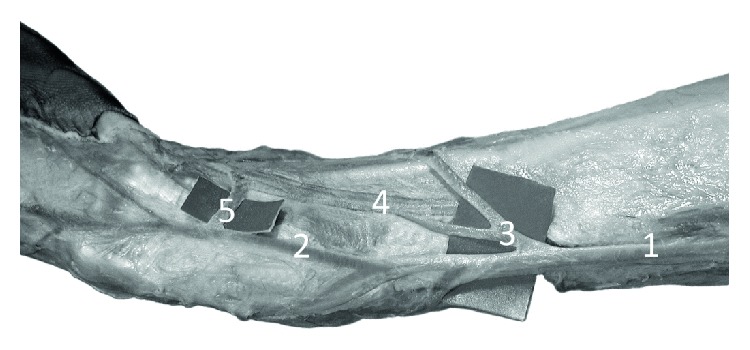
Specimen of the distal forearm and carpal region of the left forelimb of Anubis baboon (*Papio anubis*)—lateral view. The origin of the cephalic vein and perforators with the radial veins at the level of the carpus. 1—cephalic vein, 2—origin of the cephalic vein (a fusion of the dorsal digital vein from the radial side of the index finger, the dorsal digital vein of the thumb and with dorsal metacarpal vein of the second interosseous space), 3 and 5—perforating veins, 4—deep (radial) veins and the radial artery.

**Figure 2:**
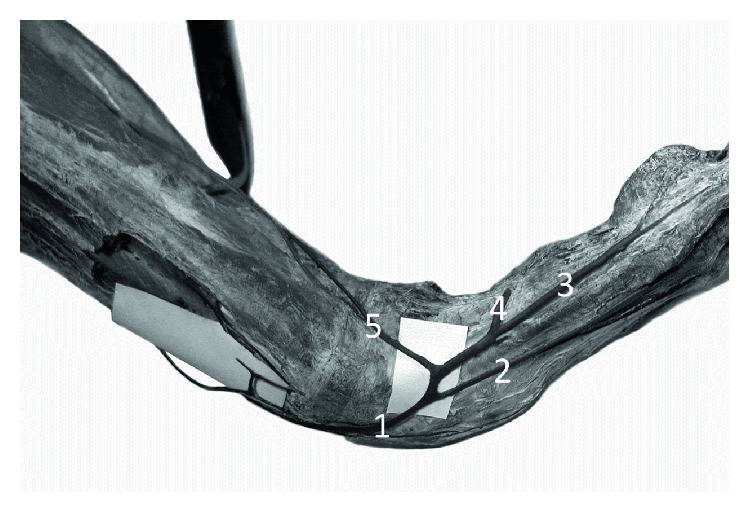
Specimen of the distal forearm and carpal region of the left forelimb of Anubis baboon (*Papio anubis*)—medial view. 1—dorsal venous arch, 2—fourth dorsal digital vein, 3—dorsal digital vein of the smallest digit, 4—perforating vein to the veins accompanying the superficial palmar arch, 5—perforating vein to the veins accompanying the ulnar artery.

**Figure 3:**
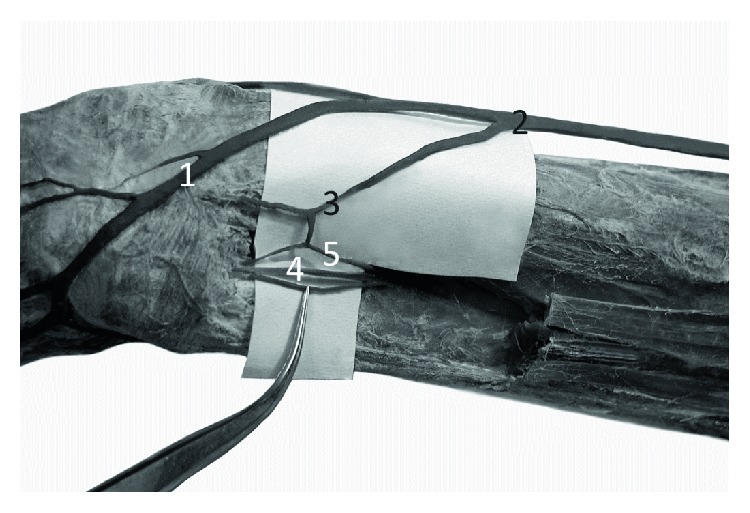
Specimen of the distal forearm and carpal region of the left forelimb of Anubis baboon (*Papio anubis*)—view on the postero-lateral surface. 1—dorsal venous arch, 2—cephalic vein, 3—anastomosis with the veins accompanying the anterior interosseous artery, 4—interosseous artery, 5—one of the veins accompanying the anterior interosseous artery.

On the dorsum of the hand, all specimens of *Papio anubis* demonstrated a well-developed dorsal venous network of hand. Within this network, the dorsal metacarpal veins could be distinguished starting from the connection of the dorsal digital veins of adjacent sites of the digits. The dorsal metacarpal veins coursed initially at the corresponding interosseous metacarpal spaces. In all of the examined specimens, the cephalic vein originated by a fusion of the dorsal digital vein from the radial side of the index finger, the dorsal digital vein of the thumb and with dorsal metacarpal vein of the second interosseous space (Figure 1). In all observed types, we noted the presence of a well-developed dorsal venous arch, running obliquely on the dorsal side of the hand and opening into the cephalic vein above the styloid process of the radius (Figures 2 and 3). The arch originated as a continuation of the dorsal digital vein of the smallest digit (*digitus minimus*) and collected the blood from the dorsal venous network of hand. In addition, in all specimens, we observed a constant anastomosis between the dorsal venous network of the hand and the deep veins accompanying the ulnar artery (Figure 2). This anastomosis began from the dorsal venous arch on the medial edge of the hand, below the styloid process of ulna. Moreover, on all examined limbs, the dorsal digital vein from the ulnar side of smallest digit gave one perforating vein to the veins accompanying the superficial palmar arch (Figure 2). Dorsal metacarpal veins anastomosed with the system of deep veins of the dorsum of the hand. The venous network on the palmar side of the hand was composed of small (<0.5 mm in diameter) venous vessels.

The cephalic vein coursed along the lateral border of the forearm. The length of the cephalic vein on the forearm was 14.4 cm on average (min = 7.8 cm, max = 19.2 cm, SD = 3.2), while the external diameter of the CV measured in the middle of the forearm was on average 2.25 mm (min = 1.52 mm, max = 3.45 mm, SD = 0.54). It was characteristic that there were numerous perforating veins within the carpal region and the lower part of the forearm. The perforators were located on the antero-lateral surface of the forearm and connected the cephalic vein with deep veins accompanying the radial artery and its well-developed superficial palmar branch (Figure 1). On the postero-lateral surface of the forearm there were permanent anastomoses with the veins accompanying the anterior (cranial) interosseous artery (Figure 3). The anastomosis branched from 1.3 cm to 4.2 cm above the styloid process of the radius (mean = 2.3 cm, SD = 1.1). The cephalic vein on its further course was located on the antero-lateral surface of the forearm.

### 3.2. Arm and Cubital Fossa

**Figure 4:**
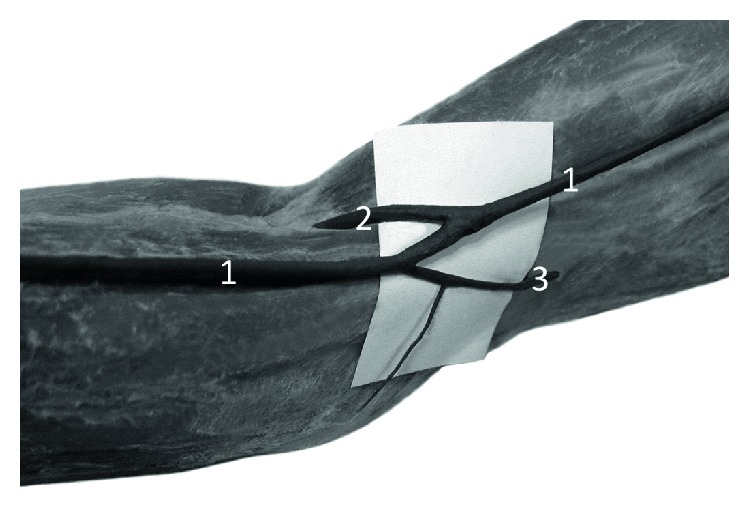
The elbow region of the left forelimb of Anubis baboon (*Papio anubis*)—antero-lateral view. The deep fascia has been preserved. 1—cephalic vein, 2—anastomosis between the cephalic vein and deep veins. The anastomosis has a characteristic, retrograde course similar to a course of median cephalic vein of human, 3—perforator connecting cephalic vein with veins accompanying the radial collateral artery.

Analogously to the forearm, within the arm of the examined specimens of the Anubis baboon, the cephalic vein was the main vessel in the superficial venous system, whereas the basilic vein was absent (Figure 4). The length of the cephalic vein measured on the arm was 14.5–19.2 cm (mean = 16.1 cm, SD = 2.7). The diameter of the CV in the middle of the arm's length ranged from 2.32 to 5.41 mm (mean = 3.72 mm, SD = 1.03).

**Figure 5:**
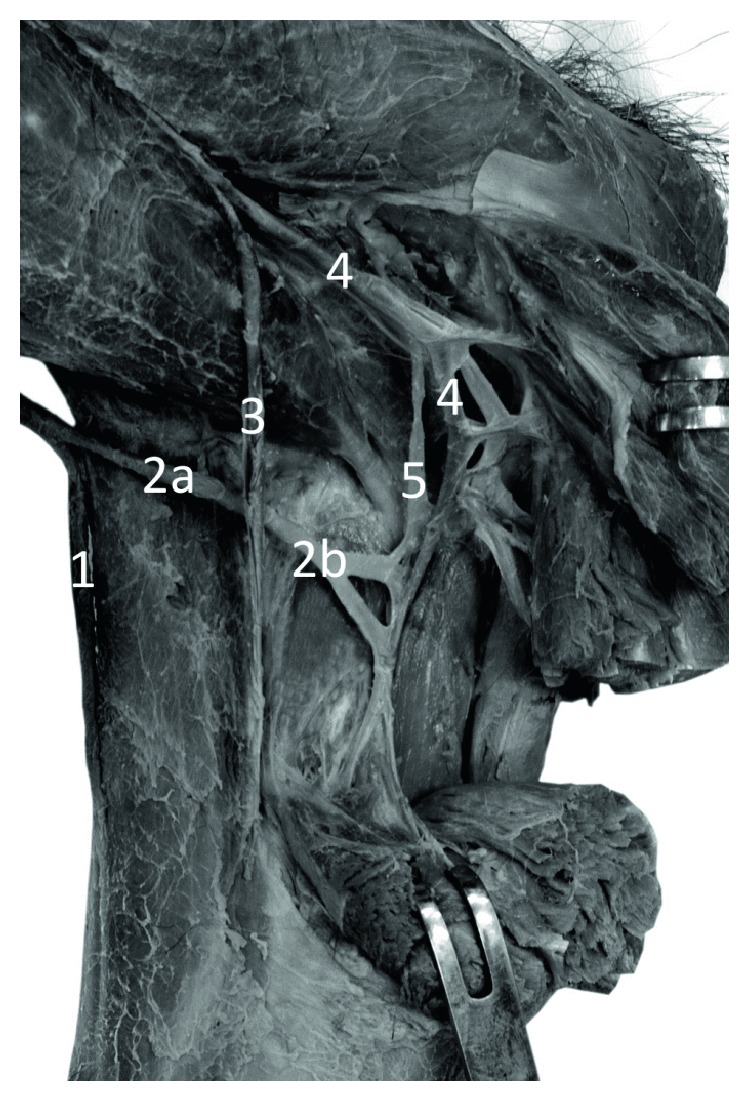
Specimen of the elbow region of the right forelimb of Anubis baboon (*Papio anubis*)—anterior view. The deep dissection. 1—cephalic vein, 2—anastomosis between the cephalic vein and deep veins. This perforator, after passing through the deep fascia (2a), connected the cephalic vein with the deep veins accompanying the radial artery and after a further short course (2b) with the veins accompanying the ulnar artery, 3—radial artery, 4—ulnar artery, 5—one of double veins accompanying the ulnar artery.

In the cubital fossa, there was a well-developed anastomosis (main cubital anastomosis) between the cephalic vein and deep veins accompanying the radial and ulnar artery. In all cases, the anastomosis showed a characteristic, retrograde course which resembled a course of median cephalic vein of the human (Figure 4). This perforator, after passing through the deep fascia, connected the cephalic vein with the deep veins accompanying the radial artery and—after a further short course—with the veins accompanying the ulnar artery (Figure 5). The diameter of this anastomosis ranged from 2.08 to 3.47 mm (mean = 2.41, SD = 0.53). Another constant perforator, located on the lateral surface of the arm, reached the veins accompanying the radial collateral artery (Figure 4). The diameter of this perforator ranged from 1.13 to 2.37 mm (mean = 1.86, SD = 0.57).

**Figure 6:**
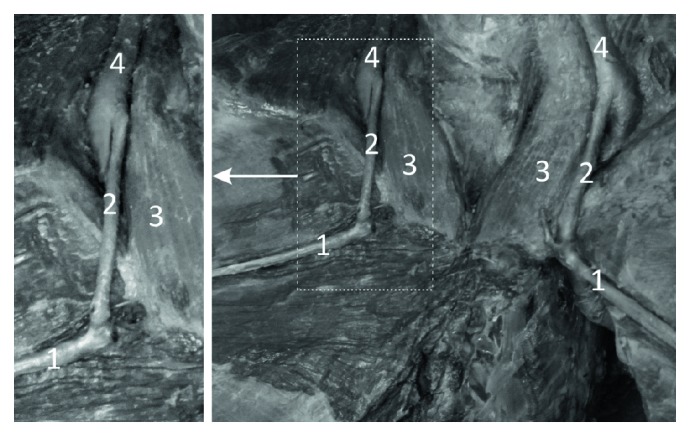
*Papio anubis*—anterior view to the shoulder and thoracic regions and the root of the neck. Persistent jugulocephalic vein (2) uniting the cephalic vein (1) and the external jugular vein (4) was exposed. 3—sternocephalicus muscle.

**Figure 7:**
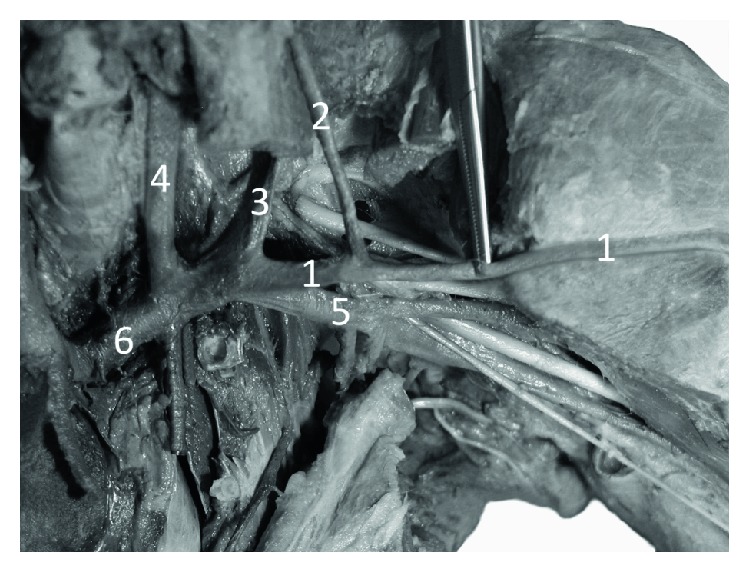
*Papio anubis*—anterior view to the thorax and the root of the neck. The deep dissection. 1—cephalic vein, 2—jugulocephalic vein, 3—external jugular vein, 4—internal jugular vein, 5—subclavian vein, 6—left brachiocephalic vein.

On its further course in the shoulder, the cephalic vein was located within the lateral bicipital groove, then entering the deltopectoral groove. The terminal segment of the cephalic vein, after passing between the clavicle and the first rib, opened into the confluence of the jugular veins and the subclavian vein (Figure 7). The diameter of the cephalic vein at the point of opening to the external jugular vein was from 2.12 mm to 7.06 mm (average 5.62 mm, SD = 1.79). It is characteristic that in all examined specimens a constant anastomosis was found between the cephalic vein and the external jugular vein; the anastomosis was identified as persistent jugulocephalic vein. The jugulocephalic vein connected terminal segment of the cephalic vein (just prior to its passage under the clavicle), crossed the clavicle from the front and then, after a short course along the lateral border of the sternocephalicus muscle, it opened into the anterolateral circuit of the external jugular vein (Figure 6). The length of the jugulocephalic vein ranged from 2.8 cm to 6.5 cm (average 4.8 cm, SD = 0.8 cm), while its diameter varied from 1.08 to 5.24 mm (mean = 2.91 mm, SD = 1.42 mm).

### 3.3. The Distribution of Valves

Bicuspid venous valves were found both in antebrachial and brachial segments of the cephalic vein of the Anubis baboon (*Papio anubis*). The valves were located in such a way that they prevented the retrograde blood flow from the proximal to the distal segments of both superficial and the deep venous systems. However, the presence of perforating veins as well as the characteristic arrangement of valves ensured free flow of the blood from the distal segments of the superficial veins to the proximal segments of the deep veins. In all examined cases, within the main cubital perforator, we found a regulatory apparatus (single venous valves) to favor the inflow of the blood to the deep veins; In particular, the backflow of the venous blood from the ulnar veins to the superficial veins was prevented by a single bicuspid valve located in the terminal segment of the main cubital perforator. Observations on venous valves distribution in the carpal region allow to assume that the blood flow between the distal segments of the deep veins and proximal segments of the superficial veins may also be possible. The data regarding distribution of venous valves within the cephalic vein of Anubis baboon was presented in Tables 1 and 2. The statistical analysis has shown that within the forearm there was a greater mean number of valves (*p* = 0.001), as well as greater intervalvular distance (*p* = 0.001). No statistically significant differences were found in this regard between the left and the right limbs (difference in number of valves between the left and right side *p* = 0.66, and for difference in intervalvular distance between the left and right limb *p* = 0.83). Moreover, no statistically significant correlation was found between the number of valves within the arm segment of the cephalic vein and the length of this segment (rho = 0.136; *p* = 0.568), as well as no statistically significant correlation was found between the number of valves within the forearm segment of the cephalic vein and the length of this segment (rho = 0,340; *p* = 0.143).

**Figure 8:**
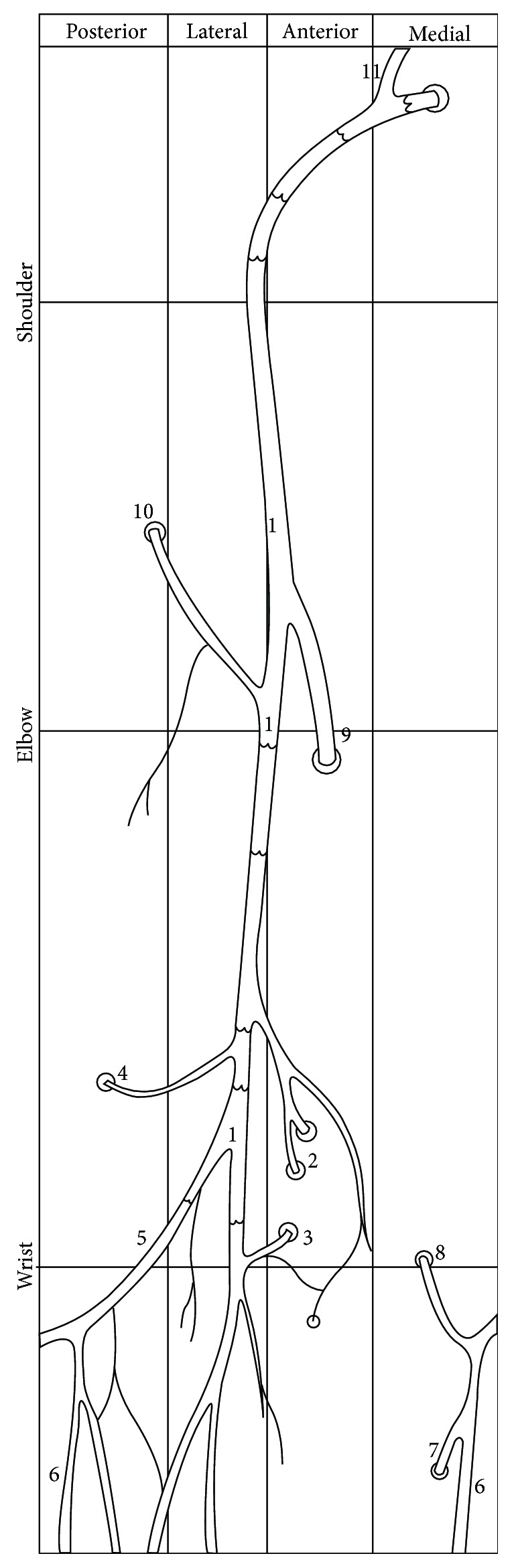
Diagram illustrating distribution of main perforators and venous valves in the superficial veins of the thoracic limb of Anubis baboon. 1—cephalic vein, 2 and 3—perforators connecting cephalic vein with the radial veins at the level of the carpus, 4—anastomosis with the veins accompanying the anterior interosseous artery, 5—dorsal venous arch, 6—dorsal digital vein of the smallest digit, 7—perforating vein to the veins accompanying the superficial palmar arch, 8—perforating vein to the veins accompanying the ulnar artery, 9—cubital anastomosis between the cephalic vein and deep (radial and ulnar) veins, 10—cubital perforator connecting cephalic vein with veins accompanying the radial collateral artery, 11—jugulocephalic vein uniting the cephalic vein and the external jugular vein.

The venous valves were arranged more evenly along the forearm segment of the cephalic vein (Figure 8). The distance between the most proximal venous valve (the last venous valve) within the forearm segment of the cephalic vein and the main cubital anastomosis (median cubital vein) ranged from 1 mm to 52 mm (mean 11.7 mm, SD = 15.6 mm). Within the arm segment of the cephalic vein we observed the valves lacking area, that involved distal half of this segment (Figure 8). The distance between the main cubital perforator (median cubital vein) and the first venous valve located across the arm segment of the cephalic vein was huge and varied from 69 to 127 mm (mean = 93.2 mm, SD = 18.8 mm). Therefore, venous valves were placed close to each other along the proximal half of the arm segment of the cephalic vein; the density of valves was high in this segment of the cephalic vein. Diagram illustrating distribution of main perforators and venous valves in the superficial veins of the thoracic limb of Anubis baboon was presented in Figure 8.

Within the jugulocephalic vein single bicuspid venous valves were found that allowed the blood to flow only in one direction—from the cephalic vein to the external jugular vein. We did not observe mono-cusp valves in our research material. Generally, valves in superficial veins of baboon were not located directly in the openings of the venous tributaries.

## 4. Discussion

Superficial veins of the upper (thoracic) limb show very high variability and diversity both in man and in other primates; regarding the number of main venous trunks, their course, as well as the origin and location of openings. In the superficial veins of *Cercopithecoidea, Pan, Gorilla*, and most of nonhuman primates, lack of significant venous trunks was observed on the medial side of the upper limb, similarly to the venous system of the Anubis baboon. Only in the human and orangutan (*Pongo*) there are two main stems—the basilic and cephalic vein, also called by some authors, regarding to nonhuman primates, respectively lateral vein and medial vein [1, 5, 23]. Observations regarding the development of the fetal veins of the upper limbs may explain the existence of diversity in the organization of the superficial veins between different species. For example, in the rat and pigs, the basilic vein is present in the earlier stages of embryonic development, and then disappears [24]. Thiranagama and his colleagues [1] put forward an interesting hypothesis, suggesting that: “in humans, and probably also in the orangutan, the possession of a basilic vein is a neotenic retention of a primitive tetrapod condition.”

The organization and variants of the dorsal venous network of hand was well documented in humans and selected nonhuman primates [7, 25, 26]. In turn, variations of the venous network on the back of the foot of the Papio anubis were also described [27]. These observations gave an analogy to our research on the arrangement of the veins of the dorsum of the hand in Papio anubis. In humans, the veins of the dorsum of hand are generally arranged in two groups located on the medial and lateral sides [7]—a similar organization of veins of the hand was observed in the Anubis baboon. However, in the venous system of the *Papio anubis*, no symmetry was observed, due to the presence of the single main venous trunk–the cephalic vein.

Typically, the cephalic vein empties into the axillary vein in some primates (*Prosimi, Anthropoidea: Ceboidea, Hominoidea-Pongo*) and in humans, or into the external jugular vein (*Cercopithecoidea, Hominoidea–Gibbon*). The cephalic vein may communicate with the external jugular vein “via a branch anterior to the clavicle” [28]. Such a branch, from the developmental point of view, may be considered the persistent jugulocephalic vein. During the development the cephalic vein empties into the venous plexus located within the neck [29]. At the 22 mm stage of embryonic development the external jugular vein emerges from the plexus and the cephalic vein opens to the external jugular vein via jugulocephalic vein [30]. Passing anterior to the clavicle, the jugulocephalic vein may persist in *Prosimia, Anthropoidea: Ceboidea, Cercopithecoidea* and in rare cases in humans [9, 21, 30–38].

Superficial and deep veins anastomose to each other through perforators piercing the deep fascia [1–7]. The role of perforating veins in providing alternative venous circulation is crucial, that some authors now include “tripartite division” of forelimb veins into “superficial, deep and perforating systems” [2, 3]. In addition, Imanischi, Nakajima, and Aiso [2, 3] indicated a three-dimensional organization of cutaneous veins considering interconnections with deep veins. In the extensive study of Thiranagama, Chamberlain, and Wood [1], perforators connecting the cephalic vein with the radial veins, occurring in the distal part of the forearm, were described by representatives of *Prosimi* (*Lemur*), *Ceboidea* (*Lagothrix* and *Alouatta*), *Cercopithecoidea* and *Anthropoidea* (in *Pan*, *Gorilla* and *Pongo* most often there were 2 perforators), while perforators connecting dorsal venous network of the hand with ulnar veins were observed in *Prosimi* (*Perodicticus*). In *Cercopithecoidea*, the presence of a perforator penetrating the extensor compartment was also noted on the posterior surface of the forearm, as in all specimens in our study (it is worth noting here that the posterior interosseous artery is less developed in baboon than in man and terminates in the forearm extensor group) [1].

In our material, numerous perforating veins were observed in the distal segment of the forearm, similarly to the report of Thiranagama, Chamberlain, and Wood [1]. Those perforators, typically less equipped with venous valves, may play an important role in regulating the direction of blood flow depending on local conditions (for example pressure and increased resistance in the system of deep veins due to muscle contraction). Also, on the forearm of the human numerous perforating veins were described [3, 4, 14, 39, 40]. Recek [10] described bidirectional flow within calf perforators with a prevailing inward direction, into deep veins. According to this author, deep and superficial veins of the conjoined vessels, as documented by nearly equal pressure curves registered simultaneously in the posterior tibial and great saphenous veins; both in varicose vein patients and in healthy people. Recek [10] stated, that the diameter of calf perforators is influenced by the intensity of saphenous reflux. This suggests that perforating veins play an important role in the dynamic regulation of blood flow between the superficial and deep veins.

The main perforators (of greater diameter) are typically located within the cubital fossa. In the studies of Thiranagama, Chamberlain, and Wood [1, 5], perforators in the area of the elbow joint were described in *Prosimi*,* Anthropoidea: Ceboidea *and* Hominoidea* (except for *Pongo*). In *Cercopithecoidea*, a perforator that runs into deep veins around the triceps was also described, which is similar to our observations regarding the Anubis baboon. In the human, in the cubital fossa, a well-developed system of perforators was described [6, 8, 12–14, 23]. In all cases examined in our study, the main cubital perforator was found with a regulatory apparatus (single venous valves) to favor the inflow of the blood to the deep veins. This observation may indicate the major role of deep veins in venous blood drainage of some species. In some representatives of *Hominoidea* (*Pan* and *Gorilla*), large venous trunks were not observed within the arm (major superficial veins were absent within this segment of the forelimb) [1, 5].

The number and distribution of venous valves may play an important role in regulation of the flow of venous blood. Since the average number of venous valves within the cephalic vein was greater on the forearm, it could be hypothesized that the longer the segment of limb and the more distal parts of it, the strongest centrifugal forces during the motion of swinging will be experienced by such region. Thus, such segment (or part) should have the greater number of valves, which is similar to our findings. Also, as Chapple and Wood [41] conclude, “valves are orientated so that they allow the blood to flow centripetally,” which was confirmed in our research.

## 5. Conclusions

In the Anubis baboon (*Papio anubis*), venous vessels were arranged in two main layers: superficial and deep, with both systems being connected by numerous perforators located at the level of the carpus and cubital fossa. In *Papio anubis* we observed the absence of the basilic vein; the main vessel within the upper (thoracic) limb, in the superficial venous system, was a well-developed cephalic vein. In all the cases, the cephalic vein opened into the external jugular vein. Also, in all of the examined specimens, there was an additional anastomosis connecting the cephalic and external jugular veins, i.e., persistent jugulocephalic vein located anterior to the clavicle. The average number of venous valves within the cephalic vein and the intervalvular distance was seen to be greater on the forearm.

## Figures and Tables

**Table 1:** The data regarding intervalvular distance within the cephalic vein (CV) of Anubis baboon.

			Arm	Forearm
Intervalvular distance [mm]	Mean		21,9	36,4
	95% confidence interval for the average	Lower limit	18,0	31,2
		Upper limit	25,9	41,5
	5% trimmed mean		19,8	35,1
	Median		18,5	36,0
	Standard deviation		12,7	18,6
	Minimum		10,2	4,1
	Maximum		74,3	98,2

**Table 2:** The data regarding number of venous valves within the cephalic vein (CV) of Anubis baboon.

		Number of valves on the arm	Number of valves on the forearm
Mean		2,95	3,65
95% confidence interval for the average	Lower limit	2,62	2,95
	Upper limit	3,27	4,35
5% trimmed mean		2,94	3,72
Median		3,00	4,00
Standard deviation		0,69	1,49
Minimum		2,00	1,00
Maximum		4,00	5,00
*p* in Shapiro–Wilk test		0,001	0,001

## Data Availability

The data used to support the findings of this study are available from the corresponding author upon request.
